# Delayed tracheal extubation after cardiac surgery due to cardiogenic ventilator auto-triggering: a case report

**DOI:** 10.1186/s40981-021-00458-4

**Published:** 2021-07-12

**Authors:** Daiki Takekawa, Satoshi Uchida, Kazuyoshi Hirota

**Affiliations:** Department of Anesthesiology, Hirosaki University Graduate School of Medicine, 5 Zaifu-cho, Hirosaki, 036-8562 Japan

**Keywords:** Ventilator auto-triggering, Cardiogenic oscillation, Delayed tracheal extubation

## Abstract

**Background:**

Ventilator auto-triggering is associated with poor outcomes. Herein, we present a case of delayed tracheal extubation after cardiac surgery due to cardiogenic auto-triggering.

**Case presentation:**

A 73-year-old male with chronic constrictive pericarditis underwent radical pericardiectomy. After confirming hemodynamic stability, we conducted spontaneous breathing trial (SBT) with a flow-trigger sensitivity of 1 L/min. As his respiratory rate (RR) increased to more than 60 breaths/min and tidal volume decreased to less than 100 mL, this SBT was considered a failure. Next morning, SBT was reperformed and the result was unchanged. However, we noticed that his heart rate and RR were the same and suspected auto-triggering caused by cardiogenic oscillations. We changed ventilator mode from flow triggering to pressure triggering of −2 cmH_2_O and he was uneventfully extubated.

**Conclusion:**

We experienced a case of delayed tracheal extubation after cardiac surgery due to cardiogenic auto-triggering. Auto-triggering can be reduced by changing ventilator trigger mode.

## Background

Ventilator auto-triggering, a type of patient-ventilator dyssynchrony, is an inappropriate triggering of a ventilator breath in the absence of spontaneous inspiratory effort. This phenomenon can be caused by circuit leaks, water condensation in the circuit, or cardiogenic oscillations [1]. Herein, we present a case of delayed tracheal extubation after cardiac surgery due to ventilator auto-triggering caused by cardiogenic oscillations.

## Case presentation

**Fig. 1:**
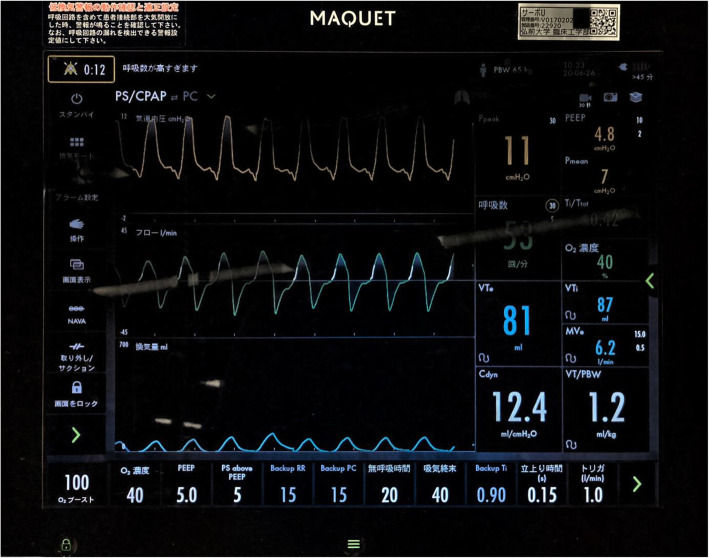
The ventilator display during the first spontaneous breathing test with flow triggering. Inspiratory pressure (up), flow (middle), and tidal volume (bottom) waveforms indicate high respiratory rate (53/min) with small tidal volume (87 ml)

**Fig. 2:**
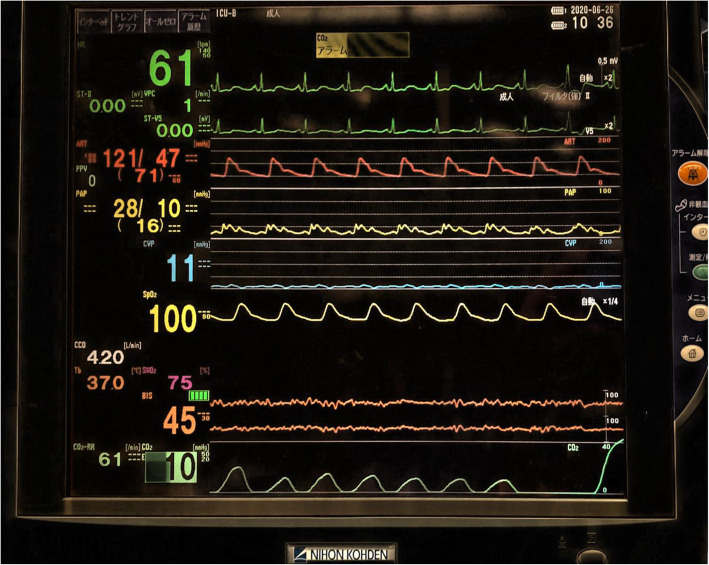
The patient monitor display during the second spontaneous breathing test with pressure triggering. From top to bottom, heart rate, blood pressure, pulmonary artery pressure, central venous pressure, saturation of percutaneous oxygen, cardiac output, body temperature (left) and mixed venous oxygen saturation (right), bispectral index, and respiratory rate (left) and end-tidal carbon dioxide (right). Heart rate and respiratory rate were the same at 60/min

**Fig. 3:**
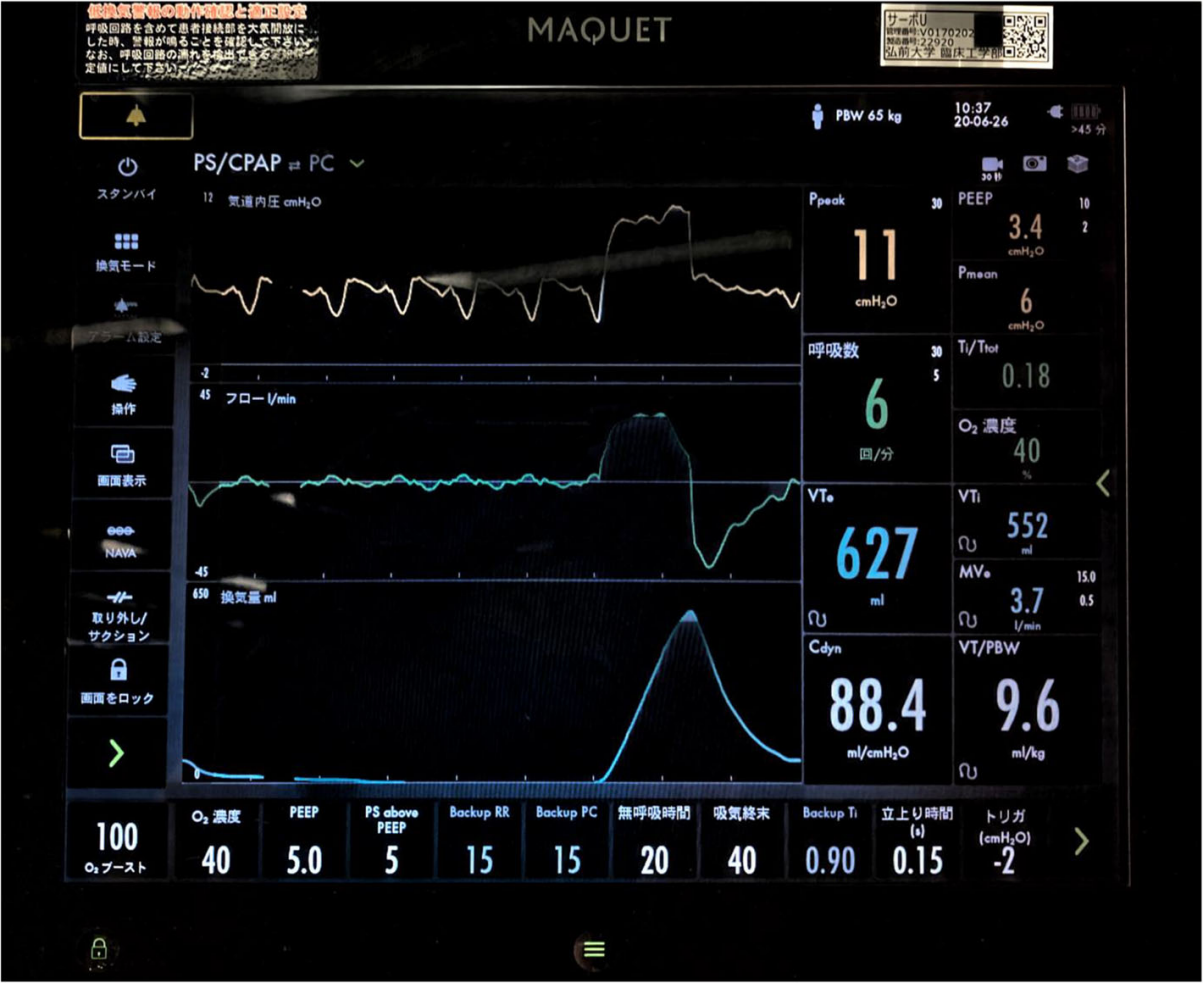
The ventilator display during the second spontaneous breathing test with pressure triggering. Inspiratory pressure (up), flow (middle), and tidal volume (bottom) waveforms indicate low respiratory rate (6/min) with large tidal volume (627 ml)

The patient was a 73-year-old male (height, 169 cm; weight, 71 kg) with chronic constrictive pericarditis. He underwent radical pericardiectomy and was postoperatively transferred to the intensive care unit (ICU) under propofol sedation with tracheal intubation. Postoperative observation was conducted under continuous intravenous infusion of propofol (1 mg/kg/h), fentanyl (20 μg/h), and dexmedetomidine (0.4 μg/kg^/^h) while maintaining hemodynamic stability. His ventilator settings on synchronized intermittent mandatory ventilation (pressure control) [SIMV(PC)] + pressure support ventilation (PSV) were as follows: a driving pressure of 15 cmH_2_O, support pressure of 15 cmH_2_O, respiratory rate (RR) of 12 breaths/min, fraction of inspiratory oxygen (FIO_2_) of 40%, positive end-expiratory pressure (PEEP) of 5 cmH_2_O, and flow-trigger sensitivity of 1 L/min. After confirming hemodynamic stability, we decreased driving pressure and support pressure to 10 cmH_2_O before starting a spontaneous breathing trial (SBT). Then, his spontaneous breaths were triggered 5 breaths/min and tidal volume was more than 700 mL when spontaneous breaths were triggered. Although RR was low due to relative deep sedation, the patient’s other status fulfilled the criteria for starting a SBT. In addition, we thought that if a SBT was started, his PaCO_2_ became higher and his RR would increase. We conducted a SBT with a PSV of 5 cmH_2_O, PEEP of 5 cmH_2_O, FIO_2_ of 40%, and a flow-trigger sensitivity of 1 L/min, and discontinued the continuous intravenous infusion of propofol. Subsequently, his RR increased to more than 60 breaths/min and tidal volume decreased to less than 100 mL (Fig. 1); this SBT was considered a failure and the prior SIMV(PC) +PSV ventilator settings were restored. The next morning, SBT was reperformed, and the result was the same as the day before. This time, however, we noticed that his heart rate (HR) and RR were exactly the same (Fig. 2), at 60/min, and suspected ventilator auto-triggering caused by cardiogenic oscillations. To examine this hypothesis, we decreased flow-trigger sensitivity to 2 L/min during, but the patient’s respiratory pattern was unchanged. Next, we changed the ventilator mode from flow triggering of 1 L/min to pressure triggering of −2 cmH_2_O. After that, his RR became 6 breaths/min and his tidal volume greater than 600 mL (Fig. 3). Upon discontinuation of the anesthetic drugs, the patient promptly emerged from anesthesia and was uneventfully extubated. Following extubation, his RR was about 12 breaths/min and he could breathe deeply. The subsequent postoperative course was uneventful, and he was discharged on postoperative day 15. We obtained a written informed consent from the patient for publication of this case report.

## Discussion

We present a case of delayed tracheal extubation after cardiac surgery due to ventilator auto-triggering caused by cardiogenic oscillations. We did not recognize the auto-triggering during the first SBT because there were a few spontaneous inspiratory efforts due to relative deep sedation; hence, the cardiogenic oscillation-triggered breaths simply looked like shallow, fast breathing. However, during the second SBT, we were able to notice that his HR and RR were the same by carefully observing his vital signs; at that point, we suspected ventilator auto-triggering caused by cardiogenic oscillations. We could definitively diagnose auto-triggering by changing the trigger setting from flow-trigger to pressure-trigger. Clinicians should consider the possibility of auto-triggering caused by cardiogenic oscillations when performing a SBT before the appearance of sufficient spontaneous breathing.

Patient-ventilator dyssynchrony including auto-triggering, which is caused by circuit leaks, water condensation in the circuit, or cardiogenic oscillations, is reported to occur in 26-82% patients [2]. Cardiogenic auto-triggering tends to occur in patients with brain death and those who have just undergone cardiac surgery [3, 4]. Patients with brain death tends to have hyperdynamic cardiovascular state [5], which may cause cardiogenic ventilator auto-triggering. Indeed, auto-triggering is reported to occur more often in patients with hyperdynamic cardiovascular state after cardiac surgery [4]. If not detected, this phenomenon can cause prolonged duration of mechanical ventilation, prolonged ICU and hospital stays, and higher ICU and hospital mortality [6–8]. Thus, clinicians must be aware of this possibility, particularly in critically ill patients.

The mechanism of cardiogenic oscillation is not completely clear, but there are several possible contributory factors. Pulmonary artery pulsatility is reported to be the main cause of cardiogenic oscillations [9]. Additionally, changes in heart volume during systole and diastole may change intrathoracic pressure, which may change airway pressure or cause compression and expansion of the lung [10]. Thus, enlargement of the heart may be associated with cardiogenic oscillation. In the present case, this patient’s postoperative cardiothoracic ratio (CTR) was 67%, indicating enlargement of heart. Indeed, cardiogenic oscillations are reported to occur in patients with larger CTR values [4].

Our PubMed search could not reveal any reports describing a relationship between cardiac oscillation and chronic constrictive pericarditis or radical pericardiectomy. However, hyperdynamic cardiovascular state after radical pericardiectomy may be associated with cardiac oscillation, because, as mentioned above, cardiac oscillation tends to occur in patients with hyperdynamic cardiovascular state [4]. Indeed, this patient’s cardiac output after surgery was more than 4.2 L/min.

One way to terminate false triggering is to change ventilator settings from the very sensitive “flow trigger” mechanism to the less sensitive “pressure trigger” mechanism [11]. In the present case, we could definitively diagnose auto-triggering by changing from the flow-trigger to the pressure-trigger setting. On the other hand, clinicians have to be careful for mistriggering, which can cause prolonged duration of mechanical ventilation, when using pressure trigger. Thus, clinicians have to select which triggering setting to use according to the patient’s situation.

## Conclusion

We experienced a case of delayed tracheal extubation after cardiac surgery due to cardiogenic ventilator auto-triggering. Auto-triggering can be reduced by decreasing ventilator trigger sensitivity. As patient-ventilator dyssynchrony may affect patients’ outcomes, early recognition is extremely important for clinicians. Clinicians have to be careful of auto-triggering when performing a SBT before the appearance of sufficient spontaneous breathing.

## Data Availability

Not applicable.

## Abbreviations

ICU: Intensive care unit
SIMV: Synchronized intermittent mandatory ventilation
PC: Pressure control
PSV: Pressure support ventilation
RR: Respiratory rate
FIO_2_: Fraction of inspiratory oxygen
PEEP: Positive end-expiratory pressure
SBT: Spontaneous breathing trial
HR: Heart rate
